# Editorial: Carbohydrates: The Yet to be Tasted Sweet Spot of Immunity

**DOI:** 10.3389/fimmu.2015.00314

**Published:** 2015-06-17

**Authors:** Deirdre R. Coombe, Christopher R. Parish

**Affiliations:** ^1^School of Biomedical Sciences, CHIRI Biosciences Research Precinct, Faculty of Health Sciences, Curtin University, Perth, WA, Australia; ^2^Cancer and Vascular Biology Group, Department of Cancer Biology and Therapeutics, John Curtin School of Medical Research, The Australian National University, Canberra, ACT, Australia

**Keywords:** Siglec, sialic acid, glycan, galectin, glycosaminoglycan, hyaluronan, heparan sulfate, heparanase

Glycobiology is an expanding discipline. Nowhere is this more apparent than in our understanding of the immune response. Perhaps the title of this focused research topic should be: “Carbohydrates: *now* the sweet spot of immunity!” The revolution in thinking to embrace the “glyco” component of glycoproteins and glycolipids has been accompanied by the development of new technologies that allow the structure of many different glycans to be determined. The article by van Kooyk et al. provides an introduction to glycan analytical tools (1). These range from technically simple analyses using plant lectins combined with flow cytometry or ELISA methods to obtain clues of glycan structures, to more complex sequencing methodologies for detailed structural characterizations. Nevertheless, determining the structure of some glycans, and particularly the glycosaminoglycans (GAGs), is still extremely difficult. However, good progress is being made in this area (2).

Cell surface glycosylation is a characteristic of all living cells (3, 4), thus it is logical that glycan structures are involved in self or non-self recognition. Nevertheless, glycans have been excluded from the thinking of most immunologists. Probably a lack of appreciation of the specificity of carbohydrate–protein interactions and the diversity of glycan structures led to this outcome. Yet, it is glycan diversity that has been harnessed by microbes to coat their surfaces, and most immunogens on microbes are glycans. As pathogens developed their glycan coats their vertebrate and invertebrate hosts similarly developed molecules to recognize these structures. The idea that invertebrate lectins can recognize glycan structures on microbes, thereby facilitating microbe phagocytosis, was accepted decades ago (5), but the fundamental contribution of glycan–protein interactions to mammalian immunity was accepted only recently. Numerous molecules involved in invertebrate host defense that recognize a spectrum of glycan structures on bacteria, fungi, and other pathogens are clearly related to similarly acting proteins in modern mammals (6). The lectin pathway of complement, toll-like receptors, the pentraxin pattern recognition receptors, and the galectins all probably arose initially in invertebrates ancestors and had roles in self or non-self recognition. We now know glycans and their binding proteins contribute to all aspects of immunology. It was argued that the essential role glycan–protein binding events play in host defense and infection is the driver of glycan diversity (3, 4). The evolutionary selection pressures imposed by the need of pathogens to avoid recognition by the proteins of their host’s immune system, and for hosts to rapidly evolve glycan structures that are not sites for pathogen adhesion and infection, it was proposed, led to the conservation of glycan structural diversity (3, 4).

An appreciation of carbohydrate structural diversity is obtained when the number of genes involved in glycan biosynthesis is appreciated. van Kooyk et al. revealed that if all the genes involved in glycan biosynthesis are considered they would comprise around 3–4% of the genome (1). Although they primarily encode enzymes, co-factors, transporters, and activated sugar donors are also involved. Regulation of the expression of these genes, regulation of the activity of the different glycosyltransferases through a diverse collection of mechanisms, coupled with regulation of the expression of core proteins adds an extra dimension to glycoconjugate structural diversity (1). Given glycan biosynthetic processes, it is not surprising that glycan structures are altered in response to physiological and pathological cues, and these different structures affect the immunological outcomes of the process in which they are involved.

Dendritic cell (DC) sialic acids illustrate how glycan structures can influence the adaptive immune response. Glycans, both *N*- and *O*-linked, on glycoproteins terminate in sialic acid, and glycolipid gangliosides contain one or more sialic acids. Sialic acids shield host cells from pathogens, prevent the deposition of complement components on DCs, and interact with receptors of the Siglec and Selectin families (7). As explained by Crespo et al., the concentration of sialic acids on DCs is very high, most Siglecs binding to sialic acids on the same DC (i.e., *cis* interactions) (7). Sialidase activity releases these Siglecs allowing them to engage in interactions with sialic acids on pathogens. The balance between cell surface sialic acid and sialidases may regulate key DC functions like phagocytosis, micropinocytosis, migration, and DC–T cell interactions (7). Not all leukocytes can have these high-sialic acid levels, nor can their Siglecs signal via sialic acid in *cis* interactions. As the binding of Siglecs on eosinophils and neutrophils to antibodies or multimeric glycan ligands triggers cell death (8), yet in certain inflammatory conditions, these cell types abound; this could not happen if these cells have high-sialic acid levels that bind Siglecs in *cis* to trigger cell death. Nevertheless, inhibitory intracellular signals upon Siglec binding sialylated antigens are common, because most Siglecs have inhibitory ITIM signaling motifs and DCs may become tolerogenic if their Siglecs recognize sialylated carbohydrate antigens in tumors (7).

Dendritic cell immunogenicity is also regulated by other carbohydrate–protein interactions; the interaction of galectin-1 with DCs encourages a tolerogenic phenotype (9). Galectins are a family of β-galactoside binding lectins. Various galectin family members have been described as “regulators of immune homeostasis,” as “pattern recognition receptors,” and as “receptors for microbial adhesion and infection” (10). Often there is evidence for the same galectin having opposing functions, the question is how? Baum et al. examined the opposing roles of galectins in microbe–host interactions (11). They described how galectins can bridge specific glycans on viral and bacterial pathogens with glycans on target cell plasma membranes, to increase pathogen attachment. The outcome of galectin–pathogen interactions is not always infection; rather there are numerous examples of galectins contributing to innate and adaptive immune responses to pathogens, and some galectins have direct microbicidal activity (11). The response is dependent on the galectin, the pathogen and the host cell, with factors such as glycan density, glycan clustering, and the glycoprotein or glycolipid upon which the glycan is presented, all contributing to the context-specific outcome. Differences in the *N*-glycans of resting and activated cytolytic T lymphocytes (CTLs), with more galectin-3 ligands being present on activated CTLs, is an example where the density of a glycan structure regulates CTL function. In a galectin-3 rich milieu (e.g., a tumor), reduced motility of galectin-3 cross-linked glycoproteins on activated infiltrating CTLs could explain the decreased CTL activity within tumors (12).

Involvement in the immune response is also in the functional realm of GAGs. Simon Davis and Parish highlight the number of proteins that have heparin/heparan sulfate (HS) binding motifs within their sequences (13). Many of the possible new HS–protein interactions that they discovered may act in immune responses but this is unconfirmed. Other confirmed HS–protein interactions have clear implications for immunity; described are examples of HS–protein interactions contributing to (1) cell adhesion and migration, (2) the regulation of cytokine and chemokine functions, and (3) the sensing of tissue injury (13). The regulation, by HS, of complement pathway triggered inflammation is emphasized by two articles. Perkins et al. used molecular modeling and affinity coefficient data to develop a bivalent, co-operative model of complement factor H (CFH) binding to HS (14). They argued, mutations in either of the CFH HS binding regions that weaken binding, alters the orientation of CFH on the cell surface disrupting C3b binding and the regulation of C3b activity, with the result being inflammatory damage, whereas, Clark et al. offered the opinion that different HS structures (or “postcodes”) in the glycomatrix of different tissues determine the levels of immobilized CFH. Probably, both explanations apply and collectively they explain the disease association of CFH polymorphisms (15).

The association of GAGs with inflammation extends beyond complement pathway regulation. Chemokine–HS interactions are known to establish chemokine gradients to direct leukocytes to inflammatory sites (16); but the contribution of the HS enzyme, heparanase (Hpse), to inflammatory disease is under appreciated. Heparanase assists leukocyte migration across basement membranes by acting as a “path-maker”; however, in type 1 diabetes Hpse activity actually drives the disease process (17). Simeonovic et al. describe how within pancreatic islets there are extraordinarily high levels of HS; this HS is essential for beta-cell survival. If active Hpse degrades HS in the islet basement membrane, inflammatory mononuclear cells can enter the islet; Hpse from these cells destroys intra-islet HS, triggering beta-cell death, and destructive insulitis. The ubiquitous non-sulfated GAG, hyaluronan (HA) is also involved in inflammation. Normally, it has a very high-average molecular weight, but at sites of inflammation and tissue injury HA polymers of overlapping length and function occur. As explained by Petrey and de la Motte, HA can promote and suppress inflammation, functions that depend upon polymer length and the activities of HA-binding proteins (18). The ability of hyaluronidases to degrade HA depends on the conformation of HA chains, which is influenced by the degree and hierarchy of protein–HA interactions, both of which depend on the HA-binding proteins in the microenvironment (18). The tissue microenvironment, its carbohydrates and their binding proteins, underpins the regulation of inflammation by HA and HS in a range of diseases including type 1 diabetes (18, 19).

The contribution of HS to human immunodeficiency virus (HIV-1) infection has come of age. Connell and Lortat-Jacob indicate how the elegant design of a potential drug developed through an appreciation of the molecular events involved in HIV-1 infection of CD4+ leukocytes (20). Although the surface exposed V3 loop of the virus protein, gp120, is involve in HS binding, prior CD4 binding was found to induce a HS binding site that is also involved in binding to HIV-1’s co-receptors, CXCR4 or CCR5. The glycoconjugate drug candidate was designed to block HIV from binding to cell surface HS, its co-receptor and CD4. It is composed of a small CD4 mimetic linked to a chemically synthesized HS dodecamer (20). This glycoconjugate had strong anti-viral activity against HIV-1 regardless of its co-receptor usage, which is a major advance.

These articles highlight the contribution of glycans to different aspects of the immune response, yet this is a “taster plate” of their total contribution. Contrary to the often held view, glycan structures frequently bind proteins with quite exquisite specificity; our lack of understanding of their binding modes and the nature of the protein conformations that are recognized cause the miss-interpretation. Reductionist thinking and analyses, although useful, in isolation are unlikely to reveal the truth. Repeatedly, it is the “context,” whether the presentation of glycan motifs, or the molecules (proteins and carbohydrates) of the surrounding microenvironment, which determines the outcome of glycan–protein interactions. It is fitting that the concluding article in this series (20) describes the development of a glycan inspired potential therapeutic, because this is an area of drug discovery currently under exploited. Advances in technologies of glycan structure determination and syntheses, coupled with a more holistic approach to understanding glycan interactions with their binding partners will lead to more glycan inspired therapeutics to treat immunological diseases.

## Conflict of Interest Statement

The authors declare that the research was conducted in the absence of any commercial or financial relationships that could be construed as a potential conflict of interest.
